# Evidence of inflated exclusive breastfeeding estimates from a clinical trial in Bangladesh

**DOI:** 10.1186/s13006-018-0179-4

**Published:** 2018-08-22

**Authors:** Thomas J. Roberts, Yana E. Hoy-Schulz, Kaniz Jannat, Julie Parsonnet

**Affiliations:** 1 Department of Medicine, Massachusetts General Hospital, 55 Fruit St, Boston, MA 02114 USA; 2 Department of Medicine, Stanford University School of Medicine, Stanford, USA; 3 Environmental Intervention Unit, Infectious Disease Division, International Centre for Diarrheal Disease Research, Dhaka, Bangladesh; 4 Department of Health Research and Policy, Stanford University School of Medicine, Stanford, USA

**Keywords:** Exclusive breastfeeding, Breastfeeding promotion, Measurement error, Epidemiology, Infant nutrition

## Abstract

Suboptimal breastfeeding is a major cause of infant morbidity and mortality across the world. Inconsistent data has hampered quantification of this practice, however, limiting breastfeeding promotion efforts. As part of a clinical trial in Dhaka, Bangladesh, data was collected on breastfeeding patterns among 125 infants. Infants were ages 4 to 12 weeks (mean = 8.05, SD = 2.13) at the time of enrollment, and breastfeeding data were collected at 24 study visits during a twelve-week period. Breastfeeding status was assessed using the WHO-recommended “current status” (24-h recall) method. These data were used to calculate two measures: a longitudinal estimate of exclusive breastfeeding since birth and a simulated cross-sectional prevalence to approximate common data collection methods. Infants were then ranked based on their breastfeeding status at all study visits and grouped into quartiles and compared using hospitalization data recorded for all infants as part of the original study. These data showed large differences in estimates of exclusive breastfeeding behaviors when assessed longitudinally (8.8% exclusive breastfeeding) vs. calculating a cross-sectional prevalence (56.2% exclusive breastfeeding). Additionally, when infants were grouped by quartile of breastfeeding behavior and matched with hospitalization records, it was found that infants in the lowest quartile of breastfeeding behaviors were significantly more likely to be hospitalized than infants in the highest quartile. These results provide further evidence that current breastfeeding epidemiology studies may overestimate rates of exclusive breastfeeding. They also provide further evidence to support the significant infant health benefits from breastfeeding promotion.

Trial registration: ClinicalTrials.gov NCT01899378. Registered July 10, 2013.

## Background

Advocacy for exclusive breastfeeding is one of the most important health interventions for infants and mothers in low-income countries [1]. Despite breastfeeding promotion efforts, it is estimated that 47 million disability-adjusted life years were attributable to suboptimal breastfeeding in 2010, making it the 5th greatest cause of human morbidity among 67 measured risk factors [2]. These estimates of the disease burden due to suboptimal breastfeeding are limited by discrepancies between breastfeeding definitions and measuring methodologies. The World Health Organization (WHO) defines exclusive breastfeeding as an infant receiving “only breastmilk. No other liquids or solids are given—not even water—with the exception of oral rehydration solution or drops/syrups or vitamins, minerals or medicines” [3]. However, the WHO recommends data collection using the “current status” method, a point prevalence based on whether infant were given any complementary foods during the 24 h prior to the time of survey. Many national and international surveys collect breastfeeding data using this method.

The “current status” data collection method has been previously shown to overestimate the prevalence of exclusive breastfeeding when compared to prospectively collected data [4, 5]. However, it not feasible to collect prospective data as part of the large cross-sectional surveys frequently used to estimate national and international breastfeeding trends [6]. Additionally, the “current status” method may be less inaccurate than methods based on maternal recall since birth [7]. The aim of this study was to compare prospectively collected breastfeeding data with a simulated cross-sectional sample from the same population of Bangladeshi infants to provide further information about the discrepancies between reported breastfeeding rates and actual breastfeeding practices.

## Methods

As part of a randomized controlled trial investigating the safety and efficacy of probiotics in infants, we collected infant feeding data on 137 infants less than 6 months old [8]. The main study was conducted at the International Center for Diarrheal Disease, Bangladesh (icddr,b) in Dhaka, Bangladesh from October 2013 through August 2014. The study enrolled healthy infants between 4 and 12 weeks of age (mean age of enrollment = 8.05 weeks, SD = 2.13 weeks) from designated neighborhoods near the iccdr,b. They were randomized to one of four study groups. Three groups received probiotics at varying frequency (daily, weekly, biweekly) and the fourth group served as a control. Data from all hospitalizations during each infant’s period of enrollment were captured as part of the main study. The main study found no difference in health outcomes or breastfeeding behaviors between the study groups [8].

Data were collected at up to 24 study visits per infant over a twelve-week period. The timing and frequency of study visits was based on the study design of the original trial. At each study visit, a brief questionnaire was administered to the infants’ mother. Breastfeeding data were collected using the WHO-recommended “current status” (24-h recall) method. Infants with less than 10 study visits with breastfeeding information were excluded from this analysis. The final sample size for this analysis was 125 infants.

When categorizing each infant’s breastfeeding status at study visits, we used WHO definitions of breastfeeding behaviors [3]. Infants given only breast milk over the previous 24 h were considered exclusively breastfed. Infants given only breast milk and water over the previous 24 h were considered predominately breastfed. Infants given breast milk and any other complementary foods over the past 24 h were considered partially breastfed. The longitudinal prevalence of exclusive breastfeeding was determined using the WHO definition of “no other food or drink, not even water, except breast milk for 6 months of life.” To be considered “exclusively breastfed” longitudinally, the infant must have been given nothing but breast milk at every prior 24-h recall.

To simulate data from a cross-sectional survey, a single study visit was randomly selected for each infant and the breastfeeding status at that visit was recorded as that infant’s breastfeeding status. The metric of prevalence of exclusive breastfeeding among infants 2–5 months was chosen to approximate results reported in recent surveys and international breastfeeding epidemiology studies [2, 9].

To assess the association between breastfeeding behaviors and health outcomes in this sample, infants were ranked based on their breastfeeding status at all study visits. Each infant was assigned a value of 0 to 3 for each study visit based on their breastfeeding status (0 for no breastfeeding, 1 for partial breastfeeding, 2 for predominate breastfeeding, 3 for exclusive breastfeeding). The mean across all study visits was calculated for each infant and the infants were ranked highest to lowest based on this value. The infants were grouped based on quartile and compared using the hospitalization data.

## Results

**Fig. 1:**
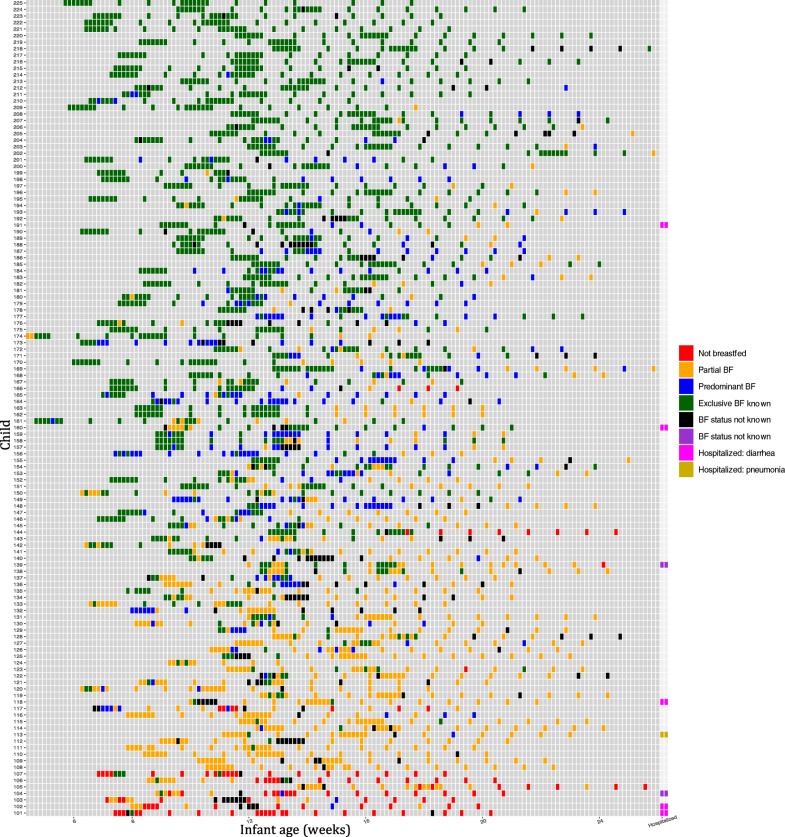
Breastfeeding status at each study visit by age of the infants. Each cell represents one study visit. The frequency of study visit was determined by the study design of the original trial. Data are listed by age of infant, not the actual date of collection. The final column indicates whether infant was hospitalized, including the reported reason for the hospitalization, during the study period

The cross-sectional sample contained 112 infants with a mean age of 13.8 weeks (SD = 4.1 weeks). The sample size was 112 because breastfeeding data was not recorded at some of the randomly selected visits. Using this sample, we calculated the prevalence of exclusive breastfeeding among children 2–5 months to be 56.2%. This number is consistent with data from the 2011 Bangladesh Demographic and Health Survey that showed an exclusive breastfeeding prevalence of 54.6% for this age group [9]. However, Fig. 1 shows the breastfeeding status for each child at each study visit, and these data revealed large discrepancies between the cross-sectional prevalence and actual breastfeeding behaviors. Of the 125 children in our sample, only 11 (8.8%) were exclusively breastfed throughout the study period. In many instances, children went through multi-day periods of partial or predominant breastfeeding before returning to “exclusive” status. The cross-sectional prevalence using the “current status” method (56.2%) was 47.4 percentage points higher than the actual (8.8%) exclusive breastfeeding prevalence calculated from prospectively-collected data.

When infants were grouped by quartile based on their breastfeeding status and compared using hospitalization records, no infants in the top quartile were hospitalized during the study period. Three infants (4.8%) in the middle quartiles, and five infants (16.1%) in the lowest quartile were hospitalized (Fig. 1; Fisher Exact Test, *p* value < 0.05). Reverse causality (ie sick infants may be unable to breastfeed) could not be completely excluded. However, the available data from safety reports and hospitalization records indicated that the causes of suboptimal breastfeeding were often independent of the infants’ baseline health status. For example, in one case breastfeeding was prematurely stopped when the mother traveled away from the home. In another case breastfeeding was not initiated because the infant was adopted.

## Conclusions

These data provide further evidence of the large discrepancy between the prevalence of breastfeeding reported using the “current status” method and actual breastfeeding behaviors observed using prospectively collected data. Based on these results and similar studies in other populations [5, 7], it appears that only a minority of women thought to be exclusively breastfeeding actually achieves this goal. Women in this study received education on the importance of breastfeeding as part of participation in the study but provided other foods nevertheless. The disconnect between education and behavioral change highlights areas for further study to improve the effectiveness of breastfeeding interventions in developing countries. They also reveal how much remains to be done to achieve infant nutrition goals.

This report was not able to assess the effects of prelacteal feeds as data about feeding practices during the infant’s first 24 h were not collected during the main study. However, breastfeeding practices through the first 6 months have been shown to be independently associated with health outcomes [10–12], and we feel that this report makes valuable contributions to the breastfeeding literature by further characterizing discrepancies between different data collection methods in a South Asian population. Additionally, this study is limited by the relatively small sample size. However, the consistency of these data with data from studies in other contexts and larger contemporaneous studies indicates they reflect true breastfeeding behaviors and accurately represent the discrepancies between data collection methods.

The discrepancies presented here are concerning. However, it is not feasible for large cross-sectional studies to prospectively collect data throughout the first 6 months of infants’ lives. These changes would limit the sample sizes of the surveys and the frequency of their administration, jeopardizing valuable sources of breastfeeding data. As the body of evidence quantifying differences between data collection methods continues to grow, future research may be able to propose correction factors that allow researchers to more reliably compare data collected using different methods and improve estimates of actual breastfeeding behaviors.

## Abbreviations

BF: breastfeeding
DHS: Demographic and Health Survey
icddr,b: International Center for Diarrheal Disease Research, Bangladesh
WHO: World Health Organization
